# High neutrophil/lymphocyte ratio is associated with poor renal outcomes in Japanese patients with chronic kidney disease

**DOI:** 10.1080/0886022X.2019.1595645

**Published:** 2019-04-03

**Authors:** Ryota Yoshitomi, Masaru Nakayama, Teppei Sakoh, Akiko Fukui, Eisuke Katafuchi, Makiko Seki, Susumu Tsuda, Toshiaki Nakano, Kazuhiko Tsuruya, Takanari Kitazono

**Affiliations:** a Department of Medicine and Clinical Science, Graduate School of Medical Sciences, Kyushu University, Higashi-ku, Japan;; b Division of Nephrology and Clinical Research Institute, Department of Internal Medicine, National Hospital Organization Kyushu Medical Center, Chuo-ku, Japan;; c Division of Nephrology, Nara Medical University, Kashihara, Japan

**Keywords:** Neutrophil/lymphocyte ratio, renal outcome, chronic kidney disease, chronic inflammation

## Abstract

**Background:** Several studies have shown that the neutrophil/lymphocyte ratio (NLR) is a marker that reflects the state of systemic inflammation. A high NLR was reported to be associated with cardiovascular events and mortality. However, little is known about the association between NLR and kidney disease progression in patients with chronic kidney disease (CKD). Therefore, the aim of the present study was to determine whether NLR is associated with renal outcomes in CKD patients.

**Methods:** This prospective observational study included 350 consecutive patients with stage 1–4 CKD treated between June 2009 and November 2016. Data were collected until June 2017. The endpoint was the composite of end-stage renal disease requiring dialysis or death. Subjects were divided into two groups according to high and low NLR levels. A Cox proportional hazards model was used to determine the risk factors for composite outcomes.

**Results:** The composite endpoint was observed in 83 patients during the median follow-up period of 31.8 months: 29 in the low NLR group and 54 in the high NLR group. Multivariable analysis showed that the high NLR group had a significant increase in the hazard ratio (HR) for composite outcomes (HR 1.67, 95% confidence interval 1.02–2.77) compared with the low NLR group.

**Conclusion:** The present study demonstrated that a high NLR was associated with poor renal outcomes, suggesting that NLR may be a useful marker for prognostic prediction in patients with CKD.

## Introduction

Chronic inflammation has an important role in the onset and progression of various diseases such as diabetes mellitus, cardiovascular disease, and chronic kidney disease (CKD) [1]. Patients with CKD tend to have elevated levels of inflammatory mediators including C-reactive protein (CRP), tumor necrosis factor-α (TNF-α), and interleukin (IL)-6 [2]. These mediators stimulate mesangial and endothelial glomerular cells and subsequently cause an increase in the production and a decrease in the degradation of the mesangial and endothelial extracellular matrix, leading to glomerular hypertension, tubulointerstitial fibrosis and renal scarring [3,4]. Because chronic inflammation is a major factor in the progression of CKD, evaluating and alleviating the extent of chronic inflammation is important to attenuate the progression of kidney dysfunction.

Several studies demonstrated that the neutrophil/lymphocyte ratio (NLR) is a potential marker for determining inflammation in cardiac and non-cardiac disorders [5–7]. It was also reported that an elevated NLR is an important predictor of mortality for patients with cardiovascular disease [8] or cancer [9]. However, NLR in patients with CKD was reported to be associated with other inflammatory markers such as IL-6 or high sensitivity-CRP [10], as well as endothelial dysfunction and cardiovascular risk [11,12]. Of note, very few studies have addressed the relationship between NLR and kidney disease progression in patients with CKD [13]. Thus, we investigated whether NLR levels were related to the decline of kidney function in patients with CKD.

## Methods

### Patients and study design

In this prospective observational study, we enrolled 350 consecutive Japanese patients with CKD who were admitted to our hospital for the evaluation and education of CKD between June 2009 and November 2016. Data were collected until June 2017. Patients with any malignancy, acute or chronic infections, chronic inflammatory disease, prescription of immunosuppressants at enrollment, acute exacerbation of CKD, and estimated glomerular filtration rate (eGFR)<15 mL/min/1.73 m^2^ at baseline were excluded from this study. All patients provided written informed consent to the protocol, which was approved by the Ethics Committee of the National Kyushu Medical Center Hospital (approval no. 09–09) and was registered at the University Hospital Medical Information Network (UMIN000017519). After discharge, all patients were followed at our hospital. The composite endpoints were end-stage renal disease requiring dialysis or death.

### Clinical and biochemical assessment

Blood samples were obtained from each patient early in the morning after an overnight fast and were analyzed for complete blood cell count, differential leukocyte count, serum creatinine, CRP, hemoglobin, serum albumin, and serum phosphorus levels. Daily proteinuria was also measured. The eGFR (mL/min/1.73 m^2^) was calculated using the new Japanese equation (194) × SCr^−1.094^×age^−0.287^×0.739 (if female) [14].

All enrolled patients were interviewed and clinically examined at presentation. Their medical histories and outpatient records were also evaluated in detail. Demographic information (age and sex), medication history, atherosclerotic risk factors (hypertension, history of smoking, dyslipidemia, and diabetes mellitus), and ischemic heart disease (IHD) at presentation were recorded for each patient. IHD was defined as a history of angina, myocardial infarction, coronary angioplasty, or coronary artery bypass surgery. Hypertension was defined as systolic blood pressure ≥140 mmHg or diastolic blood pressure ≥90 mmHg, or the current use of antihypertensive drugs. Dyslipidemia was defined as plasma triglycerides ≥150 mg/dL, plasma low-density lipoprotein cholesterol ≥140 mg/dL, plasma high-density lipoprotein cholesterol <40 mg/dL, or the use of lipid-lowering drugs based on a history of dyslipidemia. Diabetes mellitus was defined as previous or current plasma fasting glucose ≥126 mg/dL or the use of hypoglycemic agents. Cigarette smoking was evaluated as current or past. Body mass index was calculated as weight in kg divided by height in m^2^. Blood pressure was measured at three separate timepoints with the patient in a sitting position on the second day of hospitalization. The mean value of the three readings was recorded.

### Statistical analysis

Continuous data are expressed as the median (interquartile range). Categorical data are expressed as number (%). All patients were divided into two groups in accordance with the median values of the absolute NLR, absolute neutrophil count, and absolute lymphocyte count. Patients with a value of each of these three parameters that were below or above the respective median values were categorized as the low and high groups, respectively. The significance of differences between the low and high NLR groups was examined using the Chi-squared test for categorical data and the Wilcoxon rank sum test for nonparametric data. Linear regression analysis was performed to elucidate the associations between NLR and other clinical parameters.

We investigated whether high NLR, high neutrophil, or low lymphocyte group were related to the composite endpoints by applying the Cox proportional hazard model when defining low NLR, low neutrophil and high lymphocyte groups as a reference. Age, sex, traditional and nontraditional cardiovascular risk factors, and therapeutic intervention factors were selected as risk factors in the multivariable analysis. The hazard ratios (HRs) and the 95% confidence intervals (CIs) were calculated for each variable. Survival curves were estimated by the Kaplan–Meier method and evaluated by the log-rank test. We analyzed the data using the JMP10 statistics package (SAS Institute, Cary, NC). A *p* values below .05 indicated a significant difference.

## Results

The median age of the 350 patients (239 males and 111 females) in this study was 68 years (range, 20–94 years). The primary causes of renal disease were chronic glomerulonephritis (37.1%, 130 patients), hypertensive nephrosclerosis (29.7%, 104 patients), diabetic nephropathy (19.7%, 69 patients), other defined causes (11.7%, 41 patients), and unknown (1.7%, 6 patients). The clinical characteristics of the subjects are summarized according to the values below and above the median NLR value in Table 1. The NLR values ranged from 0.51 to 1.86 in the low NLR group, and from 1.87 to 5.92 in the high NLR group. For all subjects, the median eGFR value was 33.6 mL/min/1.73 m^2^ (range, 15–120 mL/min/1.73 m^2^). Twenty-one patients were CKD stage 1, 56 patients were CKD stage 2, 127 were CKD stage 3, and 146 were CKD stage 4. The high NLR group was of older age and had a higher prevalence of male subjects, smoking and IHD, compared with the low NLR group. In addition, the high NLR group had significantly higher CRP and lower hemoglobin and eGFR levels compared with the low NLR group. Table 2 shows the relationship between NLR levels and clinical parameters analyzed by linear regression analysis. In the multivariable analysis, NLR was associated with the presence of IHD, eGFR, and CRP.

**Table 1.** Baseline clinical characteristics of total patients and patients stratified by values below and above the median NLR.

Variables	Total (*n* = 350)	Low NLR (*n* = 175)	High NLR (*n* = 175)	*p*
NLR	1.87 (1.44–2.47)	0.51–1.86	1.87–5.92	
Age (years)	68 (55–77)	67 (53–75)	70 (60–78)	.02
Male (%)	239 (68)	110 (63)	129 (74)	.03
Smoking (%)	177 (51)	78 (45)	99 (57)	.02
Diabetes mellitus (%)	128 (37)	56 (32)	72 (41)	.08
Dyslipidemia (%)	246 (70)	119 (68)	127 (73)	.35
Systolic blood pressure (mmHg)	130 (118–141)	127 (117–138)	134 (120–145)	.03
Body mass index (kg/m^2^)	22.6 (20.5–25.4)	22.7 (20.4–25.7)	22.3 (20.6–24.9)	.72
Daily proteinuria (g/day)	0.8 (0.2–2.1)	0.6 (0.2–1.8)	1.0 (0.3–2.2)	.49
Hemoglobin (g/dL)	11.6 (10.0–13.0)	11.9 (10.4–13.3)	11.0 (9.5–12.7)	<.01
CRP (mg/dL)	0.09 (0.05–0.17)	0.07 (0.05–0.15)	0.10 (0.05–0.19)	.02
eGFR (mL/min/1.73 m^2^)	33.6 (22.6–56.8)	36.8 (26.6–70.3)	30.7 (20.9–43.4)	<.01
Neutrophils (/μL)	3094 (2449–3975)	2704 (2151–3212)	3643 (2909–4430)	<.01
Lymphocytes (/μL)	1703 (1279–2048)	1940 (1598–2347)	1499 (1111–1805)	<.01
Serum albumin (g/dL)	3.5 (3.2–3.8)	3.6 (3.2–3.9)	3.5 (3.2–3.8)	.31
Serum phosphorus (mg/dL)	3.5 (3.2–3.9)	3.5 (3.2–4.0)	3.5 (3.1–3.9)	.62
History of IHD (%)	57 (16)	21 (12)	36 (21)	.03

Values are expressed as the number (percent), or median (interquartile range).

NLR: neutrophil/lymphocyte ratio; CRP: C-reactive protein; eGFR: estimated glomerular filtration rate; IHD: ischemic heart disease.

**Table 2.** Relationships between NLR and clinical parameters.

	Univariable		Multivariable	
Variables	*β*	*p*	*β*	*p*
Age (years)	0.14	.01	−0.12	.12
Male	0.13	.01	0.09	.18
Diabetes mellitus	0.05	.31	−0.04	.55
Systolic blood pressure (mmHg)	0.10	.07	0.07	.25
Smoking	0.08	.14	0.00	.99
Dyslipidemia	0.03	.58	−0.01	.85
Ischemic heart disease	0.18	<.01	0.13	.02
Body mass index (kg/m^2^)	−0.01	.85	0.02	.77
Daily proteinuria (g/day)	0.02	.72	−0.11	.22
Hemoglobin (g/dL)	−0.17	<.01	−0.08	.31
eGFR (mL/min/1.73 m^2^)	−0.26	<.01	−0.23	<.01
Serum phosphorus (mg/dL)	0.00	.93	−0.01	.83
Serum albumin (g/dL)	−0.06	.30	−0.08	.35
CRP (mg/dL)	0.15	<.01	0.11	.048

NLR: neutrophil/lymphocyte ratio; eGFR: estimated glomerular filtration rate; CRP: C-reactive protein.

**Figure 1.**
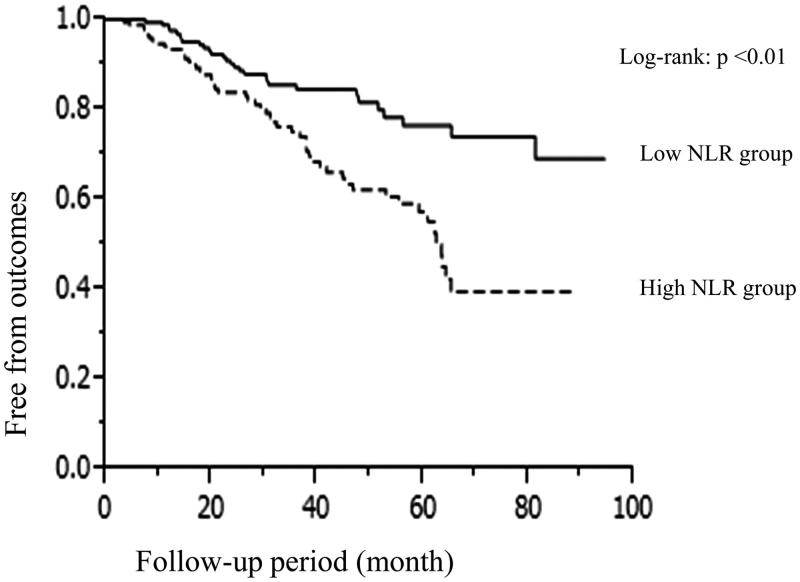
Kaplan–Meier curves with log-rank tests of freedom from composite outcomes. Data were stratified according to the values below and above the median NLR. Solid line showed low NLR group’s Kaplan–Meier curve and dashed line showed high NLR group’s one.

The median follow-up period was 31.8 months (range, 3.4–94.5 months). At the end of follow-up, the number of patients who reached composite endpoints was 83, with 29 in the low NLR group and 54 in the high NLR group. Table 3 shows the HRs for composite outcomes according to the values below and above the median values of NLR, and neutrophil and lymphocyte counts. After adjustment for demographic, traditional and nontraditional cardiovascular risk factors, and use of immunosuppressants and/or renin–angiotensin–aldosterone system inhibitors at discharge (Model 3), the high NLR group was associated with poor renal outcomes, but the high neutrophil or low lymphocyte count group was not. The Kaplan–Meier analysis demonstrated that subjects in high NLR group had a significantly higher rate of renal outcomes (Figure 1).

**Table 3.** Hazard ratios for renal outcomes of high neutrophil count, low lymphocyte count, and high NLR groups by the Cox proportional hazard model.

	Incidence rate (100 person/year)	Model 1			Model 2			Model 3		
		HR	95% CI	*p*	HR	95% CI	*p*	HR	95% CI	*p*
Low neutrophil group	6.63	(reference)			(reference)			(reference)		
High neutrophil group	9.14	1.51	0.98–2.35	.06	1.29	0.83–2.01	.27	1.13	0.71–1.81	.60
High lymphocyte group	5.73	(reference)			(reference)			(reference)		
Low lymphocyte group	9.81	1.51	0.96–2.40	.07	1.65	1.06–2.61	.03	1.14	0.68–1.93	.61
Low NLR group	4.96	(reference)			(reference)			(reference)		
High NLR group	11.10	2.22	1.42–3.54	<.01	2.06	1.31–3.30	<.01	1.67	1.02–2.77	.04

Model 1: adjusted for age and sex.

Model 2: model 1 plus adjusted for smoking, diabetes mellitus, systolic blood pressure, and dyslipidemia.

Model 3: model 2 plus adjusted for use of immunosuppressants and/or renin–angiotensin–aldosterone system inhibitors at discharge, body mass index, daily proteinuria, hemoglobin, C-reactive protein, estimated glomerular filtration rate, serum albumin, serum phosphorus, and ischemic heart disease.

NLR: neutrophil/lymphocyte ratio; HR: hazard ratio; CI: confidence interval.

## Discussion

The present study demonstrated that the high NLR group had a significantly increased risk for adverse renal outcomes, in contrast to the high neutrophil or low lymphocyte count groups, in patients with CKD stages 1–4, compared with each reference group. In addition, multivariable linear regression analysis showed that NLR was correlated with CRP, eGFR, and the presence of IHD.

It was reported that increased neutrophil counts reflected oxidative stress [15] and that lower lymphocyte counts reflected a deterioration of nutritional status [16]. However, previous reports noted that oxidative stress was involved in kidney disease progression in CKD [17] and that malnutrition was associated with adverse renal outcomes [18]. Although the present study did not show significant associations of high neutrophil or low lymphocyte counts with poor renal outcomes, the high NLR group had a predictive value for outcomes. These results suggested that NLR itself, rather than absolute neutrophil or lymphocyte counts, was a useful marker for predicting kidney disease progression.

NLR was reported to be useful for predicting mortality and cardiovascular events in patients with cardiovascular disease and malignant tumors. The main mechanism underlying the relationship between NLR and these poor outcomes was thought to be an increase in chronic inflammation, probably related to higher NLR [8,9]. The multivariable linear regression analysis in this study demonstrated that NLR levels were positively correlated with CRP levels, suggesting that high NLR reflects chronic inflammation. In addition, the current study showed that high NLR was an independent risk factor for adverse renal outcomes, independent of CRP. Previous studies reported that elevated derivatives of reactive oxygen metabolites and myeloperoxidase correlated with NLR in patients with coronary artery disease [19,20]. In CKD, chronic inflammation with increased levels of CRP, IL-6, and TNF-α, as well as oxidative stress was associated with adverse renal outcomes [1,21]. Moreover, malondialdehyde (MDA), a marker of oxidative stress, was positively correlated with NLR in active Crohn’s disease patients [22]. This suggests that MDA might have an important role in the pathogenesis of glomerulosclerosis [23]. Unfortunately, we had not examined oxidative stress or inflammatory markers other than CRP at enrollment. Nevertheless, from these findings, it might be speculated that increased oxidative stress or inflammatory markers acted as residual confounding factors in this study, and might be associated with higher NLR levels that were attributable to kidney disease progression.

In this study, multivariable linear regression analysis showed that NLR was also associated with the presence of IHD. Indeed, it was reported that high NLR levels were associated with the development of IHD in pre-dialysis [12] and dialysis patients [24]. It was also noted that the severity of IHD was related to renal outcomes [25]. Matrix metalloproteinase-9 (MMP-9), an inflammatory marker, was reported to be overexpressed in arteriosclerotic plaques and to be related to the prognosis of IHD [26]. MMP-9 is also associated with renal outcomes and renal fibrosis [27,28]. To the best of our knowledge, there has been no report directly investigating the relationship between NLR and MMP-9. However, a previous report noted that both MMP-9 and NLR were associated with left atrial remodeling in non-valvular atrial fibrillation, through a common fibrotic inflammatory process [29]. Taken together, it might be hypothesized that NLR affects renal prognosis through the same mechanism as MMP-9.

The present study had some limitations. First, the study subjects were recruited in single regional hospital; thus, the selection of patients was limited and the sample size was relatively small. Second, the study population only contained Japanese patients, and it is not yet known whether these findings can be generalized to other ethnic groups. Third, the relatively high age of this cohort might be attributable to the selection of the patients hospitalized for the evaluation and education of CKD. A larger cohort study will be needed to avoid study bias and to document more precisely the association between NLR and renal function decline in CKD patients. Finally, a single measurement of NLR may not provide sufficient accuracy for predicting the renal outcome.

In conclusion, the present study identified NLR level as an independent risk factor for kidney disease progression in patients with CKD stages 1–4. Thus, the measurement of NLR might be useful for predicting kidney disease progression in CKD patients.
